# How to create a nervous system aneurysm model in canines? ligation of the lingual artery is a simple and effective method

**DOI:** 10.3389/fphys.2023.1137564

**Published:** 2023-05-05

**Authors:** Zhengli Liu, Yuan Yuan, Rui Jiang, Boxiang Zhao, Jianping Gu, Xu He, Tao Wang, Yadong Shi, Yinghao Li, Yangyi Zhou, Guanqi Fu, Liang Chen, Maofeng Gong, Haobo Su, Jie Kong

**Affiliations:** Department of Interventional Radiology, Nanjing First Hospital, Nanjing Medical University, Nanjing, China

**Keywords:** aneurysm, nervous system, angiography, ligation, canine model

## Abstract

**Background:** The purpose of this research was to establish a safe, effective, and simple nervous system aneurysm model. This method could quickly and stably establish an exact canine tongue aneurysm model. This paper summarizes the technique and key points of the method.

**Methods**: Under the condition of anesthesia by inhaling isoflurane with a mask, we punctured the femoral artery of the canine, and the tip of the catheter was placed in the common carotid artery for intracranial arteriography. The positions of the lingual artery, external carotid artery, and internal carotid artery were identified. Then, the skin near the mandible was cut according to the positioning and separated layer by layer until the bifurcation of the lingual artery and external carotid artery was exposed. The lingual artery were then sutured with 2–0 silk sutures approximately 3 mm from the external carotid/lingual artery bifurcation. The final angiographic review showed that the aneurysm model was successfully established.

**Results**: The lingual artery aneurysm was successfully established in all 8 canines. All canines obtained a stable model of nervous system aneurysm and confirmed by DSA angiography.

**Conclusion**: We have established a safe, effective, stable and simple method to establish a canine nervous system aneurysm model with controllable size. In addition, this method has the advantages of no arteriotomy, less trauma, constant anatomical location, and low risk of stroke.

## Introduction

Nervous system aneurysms are the main cause of spontaneous subarachnoid hemorrhage. Once subarachnoid hemorrhage occurs, it will lead to high mortality and morbidity (Hughes et al., 2018). The incidence rate of nervous system aneurysms in the population is approximately 1%–5%, while the incidence of aneurysmal subarachnoid hemorrhage is about 6.67 per 100,000 people. Once ruptured and a hemorrhage of the nervous system aneurysm occurs, the mortality rate can be as high as 40% (Czorlich et al., 2017). The pathogenesis of nervous system aneurysms has not yet been clearly elucidated, and the treatment of nervous system aneurysms mainly includes surgical clipping and endovascular embolization, which can effectively avoid the occurrence of bleeding and other related complications. With the development of neuro-interventional techniques, various endovascular interventional techniques and materials are gradually applied in clinical practice. The nervous system aneurysm model was initially used to research the embolic effectiveness of coils, and later, it was also used to detect blood flow steering devices, and stent-grafts (Kallmes et al., 1999; Hans et al., 2003; Nishi et al., 2014; Kim et al., 2016). Therefore, the establishment of a simple, stable, and reliable model of nervous system aneurysm is extremely important for the research of its hemodynamics, histopathological changes, and the research and evaluation of various treatment strategies. The establishment of nervous system aneurysm models has always been complicated. Common animal model establishing methods mainly include 1. Transplant autologous veins at arterial bifurcations or arterial lateral walls (Raymond et al., 1999); 2. Ligation of common carotid artery combined with hypertension-induced aneurysm formation (Gao et al., 2008); 3. Induction of aneurysm formation in the nervous system using elastase or *ß*-aminopropionitrile (Hashimoto et al., 1987). However, these modeling methods have some disadvantages, such as the surgical trauma is large, and the modeling time is long. Meanwhile, the animals often need a long time postoperative rehabilitation, and it is difficult to accurately control the size of the aneurysm. In this research, we report the method, technical features, hemodynamic changes, histopathology, and imaging evaluation of a simple but safe and effective canine lingual aneurysm model.

## Materials and methods

### Animal

All experimental procedures were approved by the Animal Care and Use Committee at Nanjing Medical University (ACUC OF NMU). We used 8 experimental male beagles (Changzhou Beile Experimental Animal Breeding Co., Ltd., license number SCXK 2018-0007) weighing 10 kg–15 kg for the modeling of nervous system aneurysms. After completing the animal experiments, all animals were housed in the Animal Experiment Center of Nanjing First Hospital, Nanjing Medical University (license number SYXK-2021-0006).

### Method of operation

**FIGURE 1:**
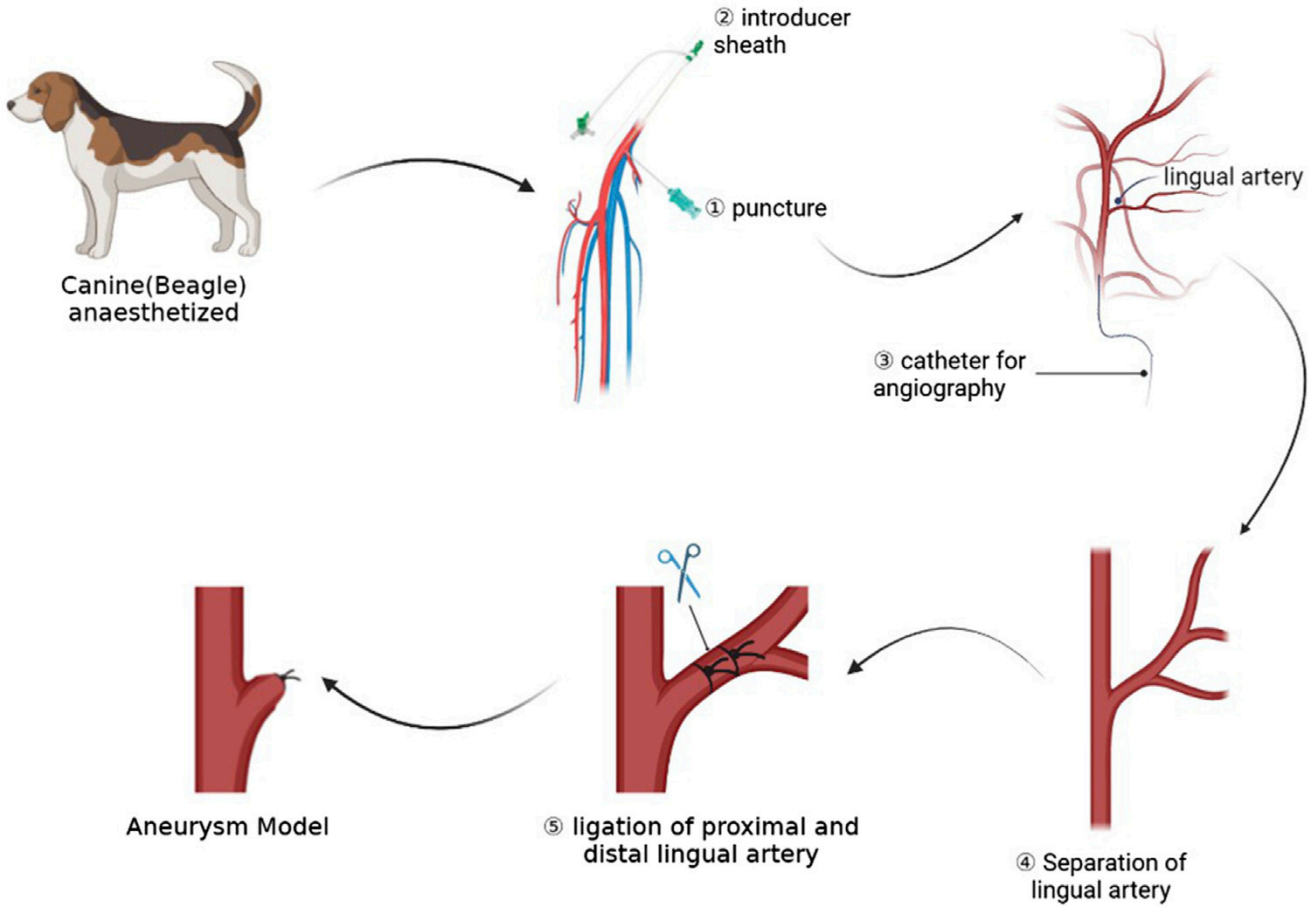
This picture shows the whole process of the experiment, including preoperative angiography, dissection of target artery, and ligation of artery to form aneurysm models.

The experiment process is shown in Figure 1. Experimental beagles in this research were anesthetized by using isoflurane (Shenzhen Ruiwode Life Technology Co., Ltd) inhalation anesthesia. Initially, the inhaled oxygen volume was adjusted to 5 L/min, and the concentration of isoflurane was 4%. We adjusted the inhaled oxygen volume after the dog’s limb muscle tone was reduced. The dosage of isoflurane during maintenance of anesthesia is 2.5%–3%. After complete anesthesia, we placed the experimental canines in a supine position and the right groin area was locally prepared, disinfected with iodophor, and covered with sterile drapes.

After we identified the right femoral artery in the dog, we used a 21 G minimally invasive needle (MeritMedical, Prelude PRO) to puncture the right femoral artery of the canine. After seeing the pulsatile ejection of blood, we introduced the micro-guide wire into the puncture kit, confirmed that the micro-guide wire was located in the abdominal aorta, and then introduced the 4 F Prelude Sheath Introducer (Merit Medical, Prelude PRO). Then, a VER135 catheter (Cordis, Miami Lakes, FL, United States of America) was used with a common guide wire (Terumo Corporation, RADIFOCUS, 0.035”) to reach the right common carotid artery of the dog, 5 cm from the bifurcation of the lingual artery and the external carotid artery. The position of the device was located in the frontal and lateral positions, and the contrast agent was injected for angiography. The position of the bifurcation of the external carotid artery and the lingual artery could be determined by angiography. During the angiography process, the metal needle of a syringe could be used to mark the lingual artery on the skin.

Then we disinfected the neck skin with iodophor and spread a sterile drape. We used a longitudinal incision to cut the skin near the needle positioning and separated the soft tissue and muscle layer by layer until the common carotid artery, external carotid artery, and lingual artery were exposed. We continued to free the lingual artery. A thread of WEGO Silk suture (non-absorbable suture, Shandong Weigao Medical Group Co., Ltd., Weihai, China) was reserved at the distal ends of the lingual artery for ligation. Then, an aneurysm clip (Shanghai Medical Instruments Co., Ltd.) was used to clip the lingual artery at the distal end of the lingual artery about 3 mm from the bifurcation, and angiography was performed to confirm the size of aneurysm. At this time, the aneurysm clip was released, and the lingual artery was ligated at the location of the aneurysm clip. When ligating, we made sure that the knot is firm and not loose. Finally, we performed the angiographic review to confirm the location and shape of the aneurysm. If the size and location of the aneurysm are not ideal, the shape and size of the aneurysm could be adjusted by adjusting the position of the line ligation.

After the surgical operation, the muscles, fascia, and skin of the canines were sutured layer by layer using simple interrupted sutures. At the same time, we disinfect the skin at the incision with iodophor and protect it with a dressing. All experimental canines were given oral aspirin (Bayer Healthcare Manufacturing Srl) 100 mg/day for 3 consecutive days after the operation to prevent platelet aggregation.

### Angiographic review evaluation

After completing the modeling of the nervous system aneurysm, we performed an angiographic re-evaluation of the nervous system aneurysm in 8 canines under anesthesia 14 days after the operation. During the angiography, a VER135 catheter was used. The tip of the catheter was located in the right common carotid artery, approximately 5 cm below the junction of the lingual artery orifice, and a contrast agent was injected for angiographic examination. The flow rate of the contrast agent was 2 mL/s, the total amount was 6 mL, and the angiography was used to evaluate the aneurysm. The parameters recorded by angiography mainly include the location and shape of the aneurysm, the width of the aneurysm neck and the height of the aneurysm.

### Digital subtraction angiography imaging

Immediately after the aneurysm modeling was completed, aneurysm-related parameters, including aneurysm neck width, aneurysm diameter, and aneurysm height, were measured and recorded. The measurement-related parameters mainly included the preoperative diameter of the lingual artery, the width of the aneurysm, the height of the aneurysm, and the diameter of the aneurysm neck at the time of reexamination. Other related operations and the location and shape of the aneurysm can be further evaluated by using three-dimensional multi-angle reconstruction in time.

After the operation, we used the SIEMENS Syngo workstation for image post-processing and selected the angiographic images immediately after modeling and 14 days respectively. We measured the angiographic images using the length measurement tool that comes with the SIEMENS Artis Zee post-processing workstation and Syngo iFlow VC21. The diameter of the bilateral common carotid arteries was measured after the ruler was calibrated. Syngo iFlow VC21 calculated the time interval from the acquisition of the image to the peak value of the gray value of each point based on each pixel value of the DSA angiography image, that is, the time-to-peak (TTP) to evaluate the speed of local blood flow. The values of TTP were measured at the common carotid artery 2 cm proximal to the lingual artery, the bifurcation of the lingual artery and the external carotid artery (the aneurysm cavity during re-examination), and the 2 cm distal internal carotid artery. At the same time, the preoperative neck was measured. The TTP value of the distal 2 cm of the internal artery was compared to compare the hemodynamic changes. The built-in point delineation of the region of interest (ROI) area in iFlow ensures that the size of the ROI area remains the same.

### Anatomy and histology

After the angiographic evaluation, the dogs were sacrificed by injection of potassium chloride and dissected. The aneurysm modeling site was separated and exposed. The aneurysm and parent artery were obtained, and the largest longitudinal section of the aneurysm and cross-section of the parent artery was sliced. Pathological and immunohistochemical examinations were carried out on the tissues. Meanwhile, the morphology of the aneurysm cavity and the changes in vascular endothelium were observed. We used CaseViewer 2.4 (3DHISTECH Ltd, Budapest) software to observe the general morphology and internal structure of the aneurysm. At the same time, the expression values of *a*-SMA, CD31, and vWF were measured. The main observation structure of the pathological section is the largest longitudinal section of the aneurysm. Image-Pro Plus 6.0 (Media Cybernetics, Inc., Rockville, United States of America) was mainly used for immunohistochemistry to measure the integrated optical density (IOD) value of each parameter in experimental canines’ expression parameters. Analytical evaluations were performed on all canines.

### Statistical evaluation

Statistical analysis was performed using SPSS 22.0 software (SPSS, Inc., Chicago, IL). The preoperative and postoperative tumor neck width, tumor size, and hemodynamic parameters were compared, and a *t*-test was used to analyze the data. *p* < 0.05 was considered statistically significant.

## Results

### Surgery and DSA of nervous system aneurysm

**FIGURE 2:**
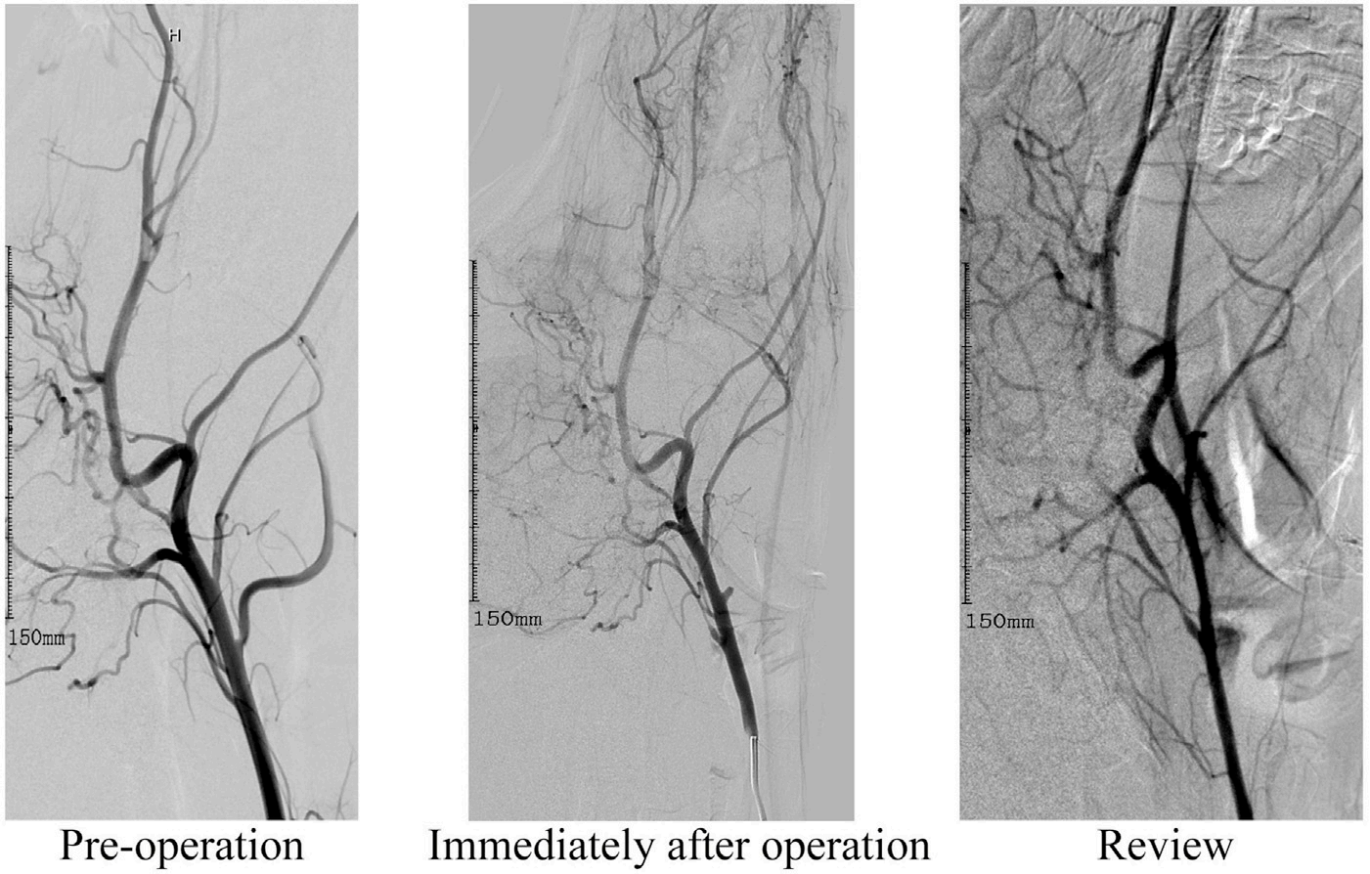
This image depicts angiographic review images before surgery, immediately after lingual artery modeling, and at the 14-day observation endpoint review.

All 8 canines in this study completed the establishment of nervous system aneurysms and angiographic review 14 days after surgery. Immediately after the ligation, angiography showed no rupture of the aneurysm and dissection of the parent artery. All dogs survived successfully to the end point of observation. After ligation of the lingual artery, the canines developed salivation, but there was no disordered eating. There were no abnormal events such as aneurysm rupture, aneurysm occlusion, and parent vessel occlusion. The diameters of the aneurysm neck and body were measured 3 times using the SIEMENS Syngo workstation, and the average value was recorded. The diameter of the lingual artery ligation of the dog before surgery was 3.17 ± 0.83 mm. The diameter of the aneurysm neck was 4.11 mm ± 0.71 mm and 4.07 mm ± 0.65 mm immediately after the operation and at the re-examination respectively. While the height of the aneurysm immediately after the operation and at the re-examination was 4.70 mm ± 0.65 mm and 4.84 mm ± 0.75 mm. The difference is not statistically significant. The results confirm that the intracranial aneurysm model established by arterial ligation is effective, stable, and reliable. The detailed data related to this model are listed in Table 1. In this study, DSA was used for contrast-enhanced evaluation and observation, and the resulting contrast-enhanced images are shown in Figure 2.

**TABLE 1:** Relevant data measured by DSA^a^.

No.	Diameter of lingual artery before operation/mm	Diameter of aneurysm neck/mm	Aneurysm height/mm	Diameter of aneurysm neck when examined/mm	Aneurysm height when examined/mm
967	3.08	3.70	4.31	4.31	4.59
847	2.00	3.54	4.71	4.62	4.92
2088	2.16	5.39	5.85	4.62	6.01
0,302	4.31	4.62	4.00	4.55	3.79
0303	3.08	3.54	4.62	2.77	4.88
2,692	4.06	4.35	5.39	4.22	5.77
1,520	3.70	4.45	4.72	4.02	4.60
766	2.97	3.31	4.00	3.47	4.14

^a^ The diameter of DSA, is determined by SIEMENS, syngo workstation.

### Gross anatomy and pathology

**FIGURE 3:**
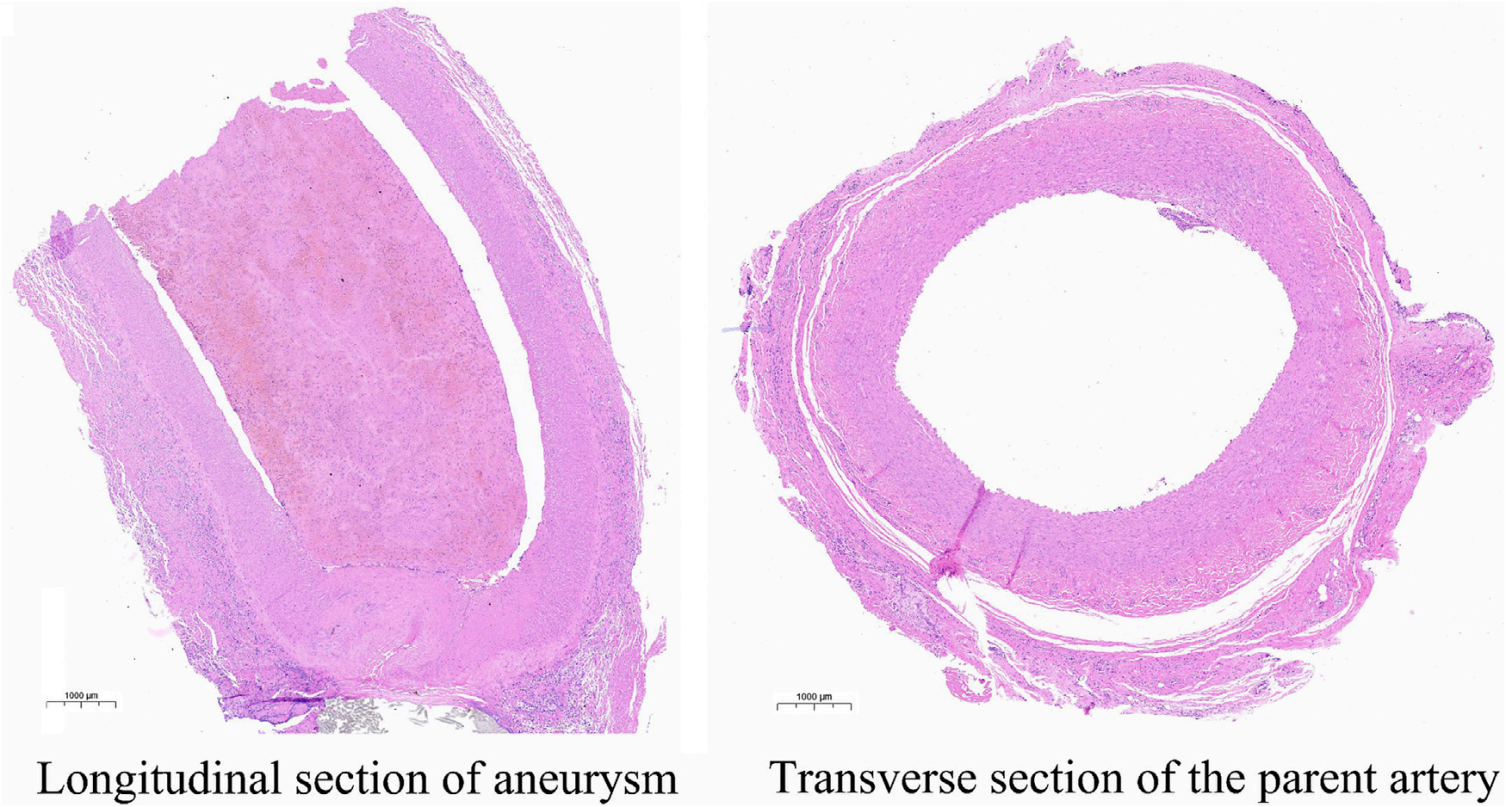
This image describes the HE pathological images of the longitudinal section of the aneurysm and the transverse section of the parent artery.

**FIGURE 4:**
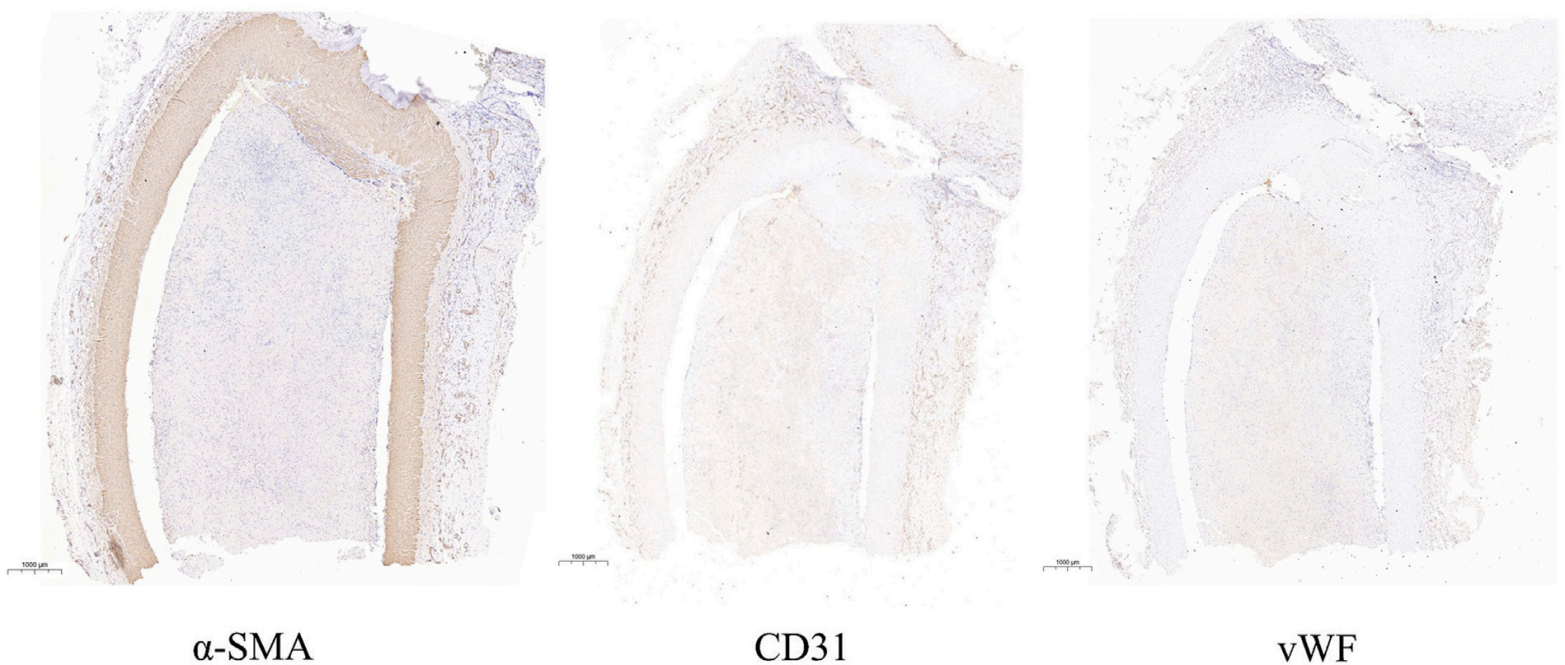
This image indicates the expressions of *a*-SMA, CD31 and vWF in the longitudinal section of aneurysm.

Animals that need to be modeled were sacrificed at the end point of observation in 30 days. After separating the aneurysm and the parent blood vessel, the general shape and internal structure of the aneurysm were observed, and the height of the aneurysm was measured 3 times. The results show that the aneurysm height and diameter of all experimental canines are 4.51 mm ± 0.60 mm, not statistically different from the images shown on angiography. The pathological section showed that the aneurysm was normal spherical, and immunohistochemistry showed that *a*-SMA, CD31, and vWF were highly expressed in the endothelium of the aneurysm. The expression parameters of the IOD value measured by Image-Pro Plus in the experimental canines were as follows: 141.35 ± 1,642.70, 125.45 ± 1,289.33 and 203.59 ± 2,870.30. This indicates the loss of the internal elastic lamina, disruption of the media and the proliferation of endothelial cells. The specific gross pathological and immunohistochemical images are shown in Figure 3 and Figure 4.

### Hemodynamic assessment of aneurysm models

**FIGURE 5:**
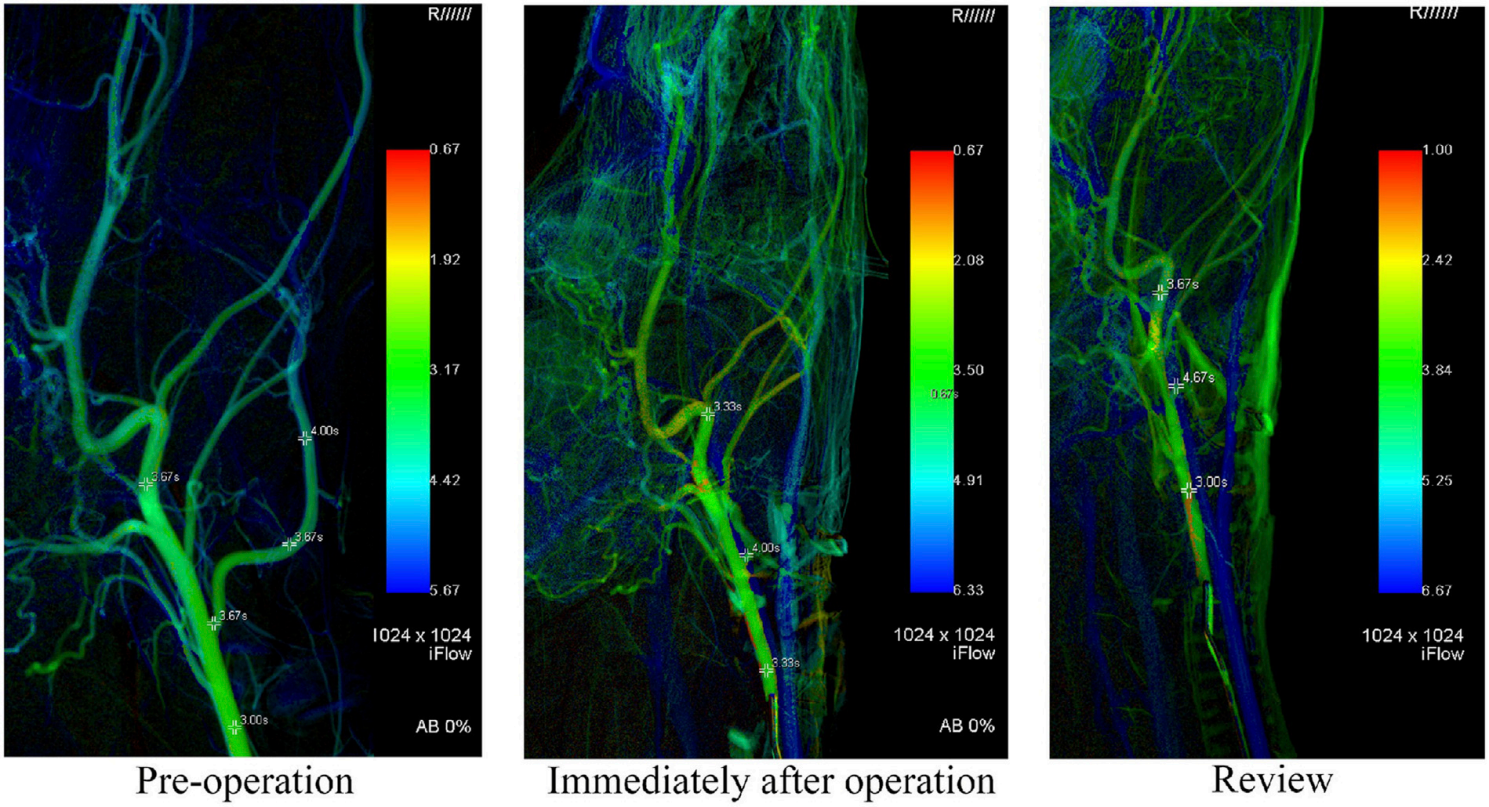
In this figure, iFlow is used to show the changes of hemodynamics. It can be seen that there is no significant impact on the distal blood flow of the parent artery after modeling.

Syngo iFlow VC21 was used to calculate the TTP value of each observation point immediately after aneurysm modeling and during the aneurysm angiography review. iFlow suggested that the TTP of the common carotid artery at 2 cm of the proximal end of the lingual artery, the bifurcation of the lingual artery and the common carotid artery, and the distal end of the internal carotid artery were 3.62 ± 0.38 s, 3.88 ± 0.30 s and 3.92 ± 0.61 s immediately after the aneurysm was modeled. The TTP values at the angiographic review after 14 days were 3.63 ± 0.42 s, 3.96 ± 0.45 s, and 3.92 ± 0.43 s, respectively. At the same time, the TTP value of 2 cm distal to the internal carotid artery before the operation was 3.46 ± 0.35 s, which was not statistically different from that after the operation. It can be considered that there was no statistical difference in the overall mean blood flow peak time before and after modeling of the lingual artery aneurysm and after modeling the observation endpoint (*p* < 0.05). The ligation of the lingual artery to establish the aneurysm model did not significantly change the peak time of the distal blood flow, which confirmed that the model was only established for the localized aneurysm model, and had no significant effect on the distal blood flow. Postoperative images of indirect assessment using iFlow are shown in Figure 5.

## Discussion

There are high mortality and morbidity rates for nervous system aneurysms (Czorlich et al., 2017). Therefore, the establishment of an aneurysm model is very important to study the pathophysiological mechanism of the aneurysm and the corresponding hemodynamic changes. A successful model of nervous system aneurysms plays an important role in the research of its occurrence, development, and later treatment process. Since the anatomy and the blood coagulation system of dogs is similar to that of humans, this experiment can more objectively simulate the basic situation of human nervous system aneurysms. The hemodynamic changes are more in line with human aneurysm disease characteristics (Dennis et al., 2009). At the same time, the canines are of moderate size and can be treated with interventional techniques, which is more suitable for the verification of various interventional treatment instruments.

In this study, 8 canine nervous system aneurysm models were successfully produced by establishing a fluoroscopy-guided canine lingual aneurysm model. All 8 models of nervous system aneurysms had no obvious signs of aneurysm neck stenosis, aneurysm occlusion, and parent artery occlusion after reexamination. In addition, the obtained aneurysm volume was stable, and the average range of the aneurysm neck and aneurysm height shown by angiography immediately after surgery and at re-examination was within 10%. At the same time, this model can easily adjust the volume and size of the aneurysm, and there is no obvious abnormal change in the shape of the aneurysm at the 14-day postoperative review. Immediately after the modeling is completed, cerebral angiography can be performed to confirm the success and stability of the modeling. Based on the experience in the quality control of nervous system aneurysm modeling accumulated in this study, we provide the following suggestions: 1) The lingual artery is located deep and originates from the external carotid artery. The mandibular angle can be used as a reference for reference. First, find the location of the internal carotid/external carotid artery bifurcation, which often requires deep exploration. After freeing the external carotid artery, the position of the lingual artery can be tracked. If necessary, fluoroscopy can be used for positioning guidance. 2) When ligating the lingual artery to establish an aneurysm model, it should be ligated from the distal end to the proximal end, so that the position of the ligation line can be adjusted more conveniently. If the ligation is performed from the proximal end to the distal end, the proximal blood vessel may be damaged, resulting in related complications such as blood vessel stenosis. 3) When ligating the lingual artery, the target blood vessel can be partially ligated at the beginning. After we identified the target blood vessel by angiography, the blood vessel can be completely ligated. 4) Aseptic technique is very important. During the operation, aseptic operation and disinfection of surgical instruments should be observed as much as possible. The incision needs to be disinfected and protected after the suture is completed to prevent infection. At the same time, antibiotics and penicillin are recommended to use for 3 days.

The use of suture ligation can directly change hemodynamics and is a relatively accurate modeling method. This method avoids the influence of uncertain factors to a certain extent, and the establishment of the animal model is more stable and reliable. This modeling method will not affect canines’ food intake, and the main complication of ligating the lingual artery is salivation. The current mainstream modeling methods for canine nervous system aneurysms mainly include transplantation of autologous veins, creation of hypertension models, induction of low-copper diet, drug-induced aneurysm formation, etc (Gao et al., 2008)- (Hashimoto et al., 1987), (Raymond et al., 1999), (Jung et al., 2016). However, these methods have different limitations. At the same time, the method of ligating the common carotid artery can be used to establish a rat model of a nervous system aneurysm, but this method is not feasible because ligation of the common carotid artery in dogs may cause ischemic stroke (Abruzzo et al., 2007). ALAN S. BOULOS et al. reported that the external jugular vein segment was anastomosed with the incision site of the external carotid lingual artery bifurcation artery to form a lateral bifurcation aneurysm (Alan et al., 2005). However, this method of autologous vein transplantation is complicated, requiring the dissociation of the external jugular vein and common carotid artery, and the surgical trauma is relatively large, which may increase the risk of infection. After the completion of autologous vein transplantation, routine feeding for at least 4 weeks is required to ensure the continuity of vascular endothelial growth. This results in the inability to ensure the controllability and stability of the aneurysm size after feeding. The aneurysm model can be established by ligating the common carotid artery and inducing hypertension by injecting cortisol and diet as well, but it has the problem of uncontrollable aneurysms (Tang et al., 2020-10). It has been reported that intracranial aneurysms can be induced by the degradation of the vascular wall caused by the use of elastase and lysyl oxidase inhibitors, as well as hypertension caused by a high-salt diet, continuous infusion of angiotensin II and ligation of the right renal artery, but the methods are complex and there are many uncontrollable factors (Patel et al., 2021). Compared with these methods, the effect of ligating the lingual artery in dogs is accurate, and the aneurysm model can be established stably and controllably with fewer long-term complications, thereby improving the success rate of model establishment. However, this method of creating aneurysms is limited in simulating vascular dilation, and we will further improve it in future research.

In summary, we have used a simple and effective method to establish a simple, stable, and controllable canine nervous system aneurysm model. This method develops definitive canine nervous system aneurysms. The model can be used for hemodynamic studies of nervous system aneurysms, providing animal experimental reference value for the treatment of aneurysms to help evaluate various treatment options and their prognosis.

## Data Availability

The original contributions presented in the study are included in the article/Supplementary Material, further inquiries can be directed to the corresponding authors.
